# Tracing Opioids Across the US: A High-Resolution Pharmaceutical Distribution Dataset

**DOI:** 10.1038/s41597-024-03534-3

**Published:** 2024-07-13

**Authors:** Ambra Amico, Luca Verginer, Frank Schweitzer

**Affiliations:** Chair of Systems Design, ETH Zurich, Weinbergstrasse 56/58, 8092 Zurich, Switzerland

**Keywords:** Business and industry, Industry

## Abstract

In the field of pharmaceutical supply chains, there is a lack of comprehensive historical data, representing a significant barrier to advancing research. To address this gap, we introduce a high-resolution dataset comprising drug packages distributed to approximately 300,000 pharmacies, hospitals, and practitioners across the US. We reconstruct 375 million distribution paths from ARCOS, a DEA-maintained database comprising half a billion shipping records between 2006 and 2014. While ARCOS tracks dyadic shipments, it does not provide information on the complete journey of single packages from manufacturers to final destinations. Our algorithm is able to reconstruct complete distribution paths from these dyadic records. The reconstructed dataset, with its high temporal and spatial resolution, offers an unprecedented view of US pharmaceutical distribution and is a valuable resource for investigating supply and distribution networks.

## Background & Summary

A quick response to shortages of essential goods, such as medicines, is crucial. Today’s supply chains span the globe, linking thousands of manufacturers, distributors, and consumers in worldwide supply networks. Fine-grained data on such large-scale networks would be valuable for researchers and policymakers to devise shortage mitigation strategies^1–3^. The challenges to do so are twofold. First, advanced methods are needed to process such data as they have spatial, temporal, and network dimensions. The second challenge is to extract valuable insights for stakeholders.

These challenges have not been addressed adequately due to a lack of comprehensive historical data. This lack primarily originates from firms’ reluctance to disclose proprietary data for fear of revealing sensitive competitive information. Consequently, the available data often lack the resolution and detail necessary for comprehensive analysis^4^.

To deal with these challenges, researchers have turned to alternative data sources as proxies for the supply network’s structure. For example Reisch *et al*.^5^, exploited mobile phone data to map Hungary’s supply network, while Dhyne *et al*.^6^ harnessed VAT tax declarations to uncover the connections among Belgian firms. Similarly Carvalho *et al*.^7^, Inoue and Todo^8^, employed survey data to study the Japanese supply network in the wake of the 2011 earthquake.

Also, many countries are instituting Pharmaceutical Track and Trace (PTTS) programs. Initiatives like the European Medicines Verification Organisation (EMVO), commencing in 2022, aim to trace pharmaceutical packages. However, the available data remains limited in scale, temporal resolution, and periods covered. They are often restricted to selected firms and temporally aggregated (e.g., over years).

We present a high-resolution dataset detailing the *distribution paths*, from manufacturing to consumption, of billions of drug packages throughout the United States. This dataset extends over nine years, from 2006 to 2014, and offers a daily resolution. We reconstructed this dataset from the “Automation of Reports and Consolidated Orders System”, ARCOS for short. The dataset is maintained by the Drug Enforcement Agency (DEA) and used to record all legal shipments of abuse-prone pharmaceuticals, especially opioids (e.g., oxycodone and hydrocodone). ARCOS contains over half a billion shipping records, now publicly available due to legal proceedings related to the US opioid crisis. However, it provides only dyadic shipment data, meaning that it records individual shipments between pairs of entities (manufacturers or distributors) and does not track the complete journey of pharmaceutical products through the distribution system. This limitation hinders various analyses, such as modeling product recalls, detecting anomalous distribution paths, and identifying alternative routes during shortages.

Here, we present our algorithm to reconstruct these *distribution paths* from the ARCOS shipping records. The algorithm enables us to trace single packages as they move from manufacturers, through distributors, and ultimately to hospitals, pharmacies, or practitioners. The reconstructed dataset that we provide offers a fine-grained and novel view of the US opioid distribution dynamics, enabling researchers to simulate potential system responses to disruptions like production halts, trade issues, or natural calamities. Notably Amico *et al*.^9^, utilized this dataset to evaluate the resilience of the opioid distribution network through a stress-test approach.

This paper is structured as follows: We begin with a detailed description of the ARCOS dataset. Next, we introduce our method for reconstructing distribution paths. Then, we discuss several descriptive statistics, followed by a validation of the reconstructed paths. The validation involves testing the core assumption of the reconstruction method, which specifies “First-in-first-out” (FIFO) as the stock management strategy for the underlying distribution process. FIFO determines the order in which batches are dispatched from stocks, ensuring that the batches received first are also processed first to avoid product expiration^10^. Lastly, we assess the stability of the reconstructed paths over time, demonstrating that they do not change abruptly, indicating a stable pharmaceutical distribution system.

## Methods

### Data source

The data source of our reconstruction algorithm is ARCOS^11^, a DEA-maintained database collecting all shipments of controlled substances in the United States. These data were recently released to the public as a result of a court order within the opioid crisis litigation^12^. Note that while ARCOS tracks various controlled substances, such as hallucinogens, only data on opioid drugs were made publicly available. Additionally, ARCOS does not include information about shipments of raw materials intended for manufacturing; it exclusively covers finished products.

We obtained the data from the SLCG consulting group, which was commissioned to analyze them for legal proceedings and provide access to the raw dataset^11^. Specifically, this dataset contains 499,534,836 shipping transactions representing all legal shipments of opioid drugs occurring in the US from 2006 to 2014. It encompasses all 50 states, six US territories, and four service lands. Only 0.01% of these transactions are international (i.e., import/export), suggesting a predominantly closed US opioid distribution system.

The transactions involve various types of entities, such as manufacturers, distributors, hospitals, and practitioners. Whether an entity is identified as a manufacturer, a distributor, or a hospital is part of the DEA classification and is indicated in the dataset under the attribute “business activity”. Then, we use the umbrella term “final distributors” to identify all entities that are neither manufacturers nor distributors and that represent the end of the distribution chain. These include hospitals, pharmacies, practitioners, and clinics. Note that ARCOS does not record sales to patients. Each entity is identified by a unique DEA number and is reported along with various information, such as company name and location; see Table 1 for an example. We count 610 manufacturers, 1,313 distributors, and 299,344 final distributors, for a total of 301,267 entities. Drugs are identified by their National Drug Code (*NDC*), an 11-digit number. This number encodes information about: the manufacturer (first 5 digits); the drug formulation, including its active ingredient, dosage form, and strength (second 4 digits); and the package size (last 2 digits). Fourteen active ingredients are listed in the raw dataset, e.g., fentanyl, hydrocodone, and oxycodone. Dosage forms include tablets, pills, liquids, bottles, powders, and skin patches. Package size indicates the amount of product in a single drug package, e.g., 50 pills or 10 tablets.

**Table 1:** Example of ARCOS transaction table.

Date	Sender	Receiver	Quantity	Sender Type	Receiver Type
2010-06-22	RM0231821	RM0270037	200	Manufacturer	Distributor
2010-07-29	RM0270037	PR0205559	10	Distributor	Distributor
2010-07-16	RO0153609	AA1680049	1	Distributor	Hospital
⋮	⋮	⋮	⋮		
2010-10-11	PR0205559	BP3701047	2	Distributor	Pharmacy

ARCOS contains primarily two types of transactions: sales and purchases, which account for 96% of all transactions. Sales transactions are shipments declared to the DEA by the shipping entities. Purchase transactions are shipments declared to the DEA by the receiving entities. Due to the double-entry bookkeeping nature, transactions are recorded by both the sender and the receiver. However, only manufacturers and distributors are required to report their transactions, while final distributors are not. Thus, shipments to final distributors (e.g., pharmacies) are only captured in the sales transactions. Therefore, we only work with sales transactions. Note that prices are not listed.

### From shipping transactions to distribution paths

Despite its extensive coverage, ARCOS lists only dyadic shipping transactions, namely shipments between pairs of distributors; it does not reveal the complete distribution path followed by drug packages. For instance, as reported in Table 1, a single transaction represents the shipment from a manufacturer to a distributor. Thus, dyadic shipments represent only one of several steps in the distribution process.

**Fig. 1:**
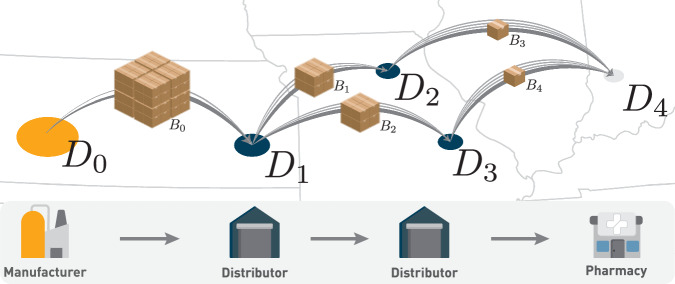
The map illustrates a package’s journey, starting from its origin at the manufacturer, *D*_0_, moving through distributors, *D*_1_, *D*_2_, and *D*_3_, before reaching its final destination at a pharmacy, *D*_4_. Arrows on the map trace this specific example, showcasing how the packages are split and distributed through various stages before reaching their destination.

A *distribution path*, instead, tracks every step of the distribution process, starting from the manufacturer, passing through multiple distributors, and ending at the final distributor (e.g., a hospital, pharmacy, or practitioner). We depict an example of a distribution path in Fig. 1. This is the path *D*_0_ → *D*_1_ → *D*_2_ → *D*_4_, starting in the manufacturer *D*_0_, passing through the distributors *D*_1_ and *D*_2_ and ending in the final distributor *D*_4_. This illustrative path has length 3, as it involves 3 distribution steps. However, generally, distribution paths may be of variable length.

To reconstruct distribution paths from ARCOS data, it’s essential to connect dyadic shipments correctly. This task is particularly challenging because packages are shipped in batches that can change in size during the distribution process. Manufacturers typically dispatch large batches, each containing multiple packages. Distributors keep these batches in stock and then repack them for further shipments^13,14^. As a result, when transactions are recorded in ARCOS, the information about the specific source batch is lost.

Thus, the fundamental challenge in reconstructing distribution paths lies in identifying the particular batch from which individual packages originated. To better illustrate the issue, consider the following situation: a distributor has two batches in stock, arriving at different times and sourced from two different manufacturers. It’s unclear which batch will be used to fulfill an order. Therefore, the distribution path can be reconstructed in two different ways, depending on which batch is used first. The decision of which batch to use first for order fulfillment is determined by the stock management strategy. This choice represents the only degree of freedom we have and forms the primary assumption of our reconstruction method.

### Stock management strategies

There are two main strategies: First-in-first-out (FIFO) and Last-in-first-out (LIFO). In FIFO, batches arriving first are dispatched first, i.e., the oldest batches are processed first. In LIFO, batches arriving last are dispatched first, i.e., the newest batches are processed first. Given that our data is on perishable goods, we assume a FIFO strategy, which is also recommended by the World Health Organization WHO^15^ in its “Good Distribution Practices”.

Although FIFO is arguably the most suitable stock management strategy for perishable goods, it remains an assumption in our method. To gain a deeper understanding of how an alternative stock management strategy might impact the properties of the reconstructed dataset, in the Technical Validation section we conduct a comparative study by contrasting FIFO with LIFO. Unlike FIFO, LIFO may cause batches to be processed quicker, but older batches will remain in stock much longer and possibly expire.

### The algorithm

The underlying idea of the algorithm is to reconstruct the distribution paths by processing the shipping transactions chronologically. We initialize an empty stock for each manufacturer or distributor in the ARCOS database, represented as *D*_*i*_. As transactions are processed, stock levels change as distributors receive and ship goods.

To illustrate the algorithm, consider the following example based on Fig. 1: on the first day *t* = 0, a batch *B*_0_ containing ten doses is dispatched from the manufacturer *D*_0_ to the distributor *D*_1_. At this point, we record the first step of the distribution path as *P*_0_ = (*D*_0_, *D*_1_) and add this information to the batch *B*_0_. The next day, *t* = 1, we observe that distributor *D*_1_ undertakes two transactions: it ships two units to *D*_2_ and five units to *D*_3_. Thus, *D*_1_ divides the batch *B*_0_ into two new batches, *B*_1_ and *B*_2_, containing two and five units, respectively. While batch *B*_1_ and *B*_2_ are dispatched to *D*_2_ and *D*_3_, we update the distribution paths *P*_2_ = (*D*_0_, *D*_1_, *D*_2_) and *P*_3_ = (*D*_0_, *D*_1_, *D*_3_) associated with *B*_1_ and *B*_2_, respectively. The next day, *t* = 3, both *D*_2_ and *D*_3_ ship a batch of 1 unit each, i.e., *B*_3_ and *B*_4_, to the pharmacy *D*_4_, a final distributor. Again, we extend the paths associated with the batches arriving at *D*_4_ and obtain the following distribution paths: *P*_4_ = (*D*_0_, *D*_1_, *D*_2_, *D*_4_) and *P*_5_ = (*D*_0_, *D*_1_, *D*_3_, *D*_4_).

After processing all transactions, we identify all distribution paths ending with a final distributor. All other disruption paths are associated with batches that are still in transit and have not yet completed their distribution journey. In the example above, this means that we only collect paths *P*_4_ and *P*_5_ as they reach the final distributor *D*_4_.

Note that as transactions are processed, the number of packages available in a given batch may be smaller than the quantity that needs to be shipped. For example, *D*_3_ needs to ship 5 units to pharmacy *D*_4_ but has only the following batches in stock: *B*_3_ with four units and *B*_6_ with two units, where *B*_3_ arrived before *B*_6_. In this case, multiple packages from different batches must be combined. Assuming the FIFO stock management strategy, we first use the four units from *B*_3_, the oldest, and then the remaining 1 unit is taken from *B*_6_, the second-oldest. This means that we obtain two distribution paths, *P*_6_ = *P*_7_ = (*D*_0_, *D*_1_, *D*_3_, *D*_4_) with different quantities (i.e., 4 and 1 units) and different timing (i.e., *P*_7_ starts later).

As illustrated above, the stock management strategy is the primary rule assumed by our algorithm. The timing and quantity of transactions are entirely determined by the data. The pseudocode for this algorithm is shown in Algorithm 1.

#### Algorithm 1

Path reconstruction algorithm.

## Data Records

### Data composition

**Fig. 2:**
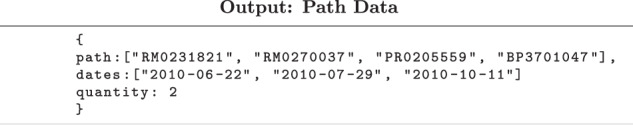
A example record of the reconstructed distribution paths. It contains: *path*, the sequence of distributors; *dates*, the shipping dates; and *quantity*, the units of products received by the final distributor.

Using the algorithm described above, we reconstruct the distribution paths of 18,672 drug products, identified by their *NDC*, and obtain 375 million paths from 2006-2014. For each product, we provide a separate JSONL file containing all its reconstructed distribution paths. For a given distribution path we report: (i) its *path*, namely the sequence of distributors traversed by packages and identified by their DEA number; (ii) the chronology of the shipping dates; (iii) and the quantity shipped to the final distributor. See Fig. 2 for an example record. The data are available at Figshare^16^. Be aware that the paths reconstructed for the early months of 2006 are less reliable due to incomplete initial stock data.

In addition to this dataset, we provide two auxiliary files: entities.csv and products.csv. The entities.csv file contains five columns detailing the business activities, city, state, and zip code of each registered DEA number. The products.csv file identifies the NDC for each shipped product, along with its active ingredient, such as hydrocodone or oxycodone.

### Descriptive

**Fig. 3:**
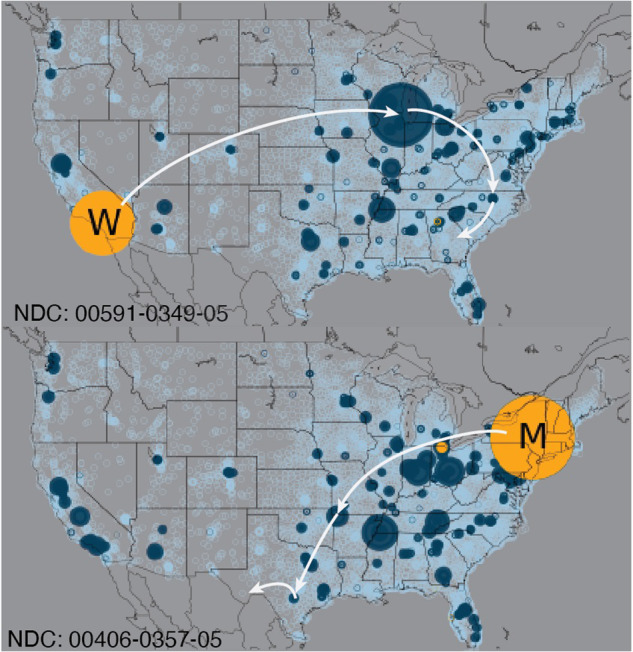
The geographic location of distributors for two packages of “Hydrocodone Bitartrate and Acetaminophen”, one produced by Watson (top) and one produced by Mallinckrodt (bottom). These two products have the highest number of transactions for hydrocodone-based products. Manufacturers are shown as yellow circles, distributors as blue circles, and the final distributors as light blue circles. The circle sizes are proportional to the outflows aggregated over the nine years. Two example distribution paths are highlighted in white.

To provide insights into the characteristics of the reconstructed dataset, we present various statistics and visualizations describing topological, temporal and geographical aspects. Figure 3 illustrates the large-scale nature of the distribution system under study. The Figure displays the spatial concentration of distributors of two hydrocodone-based products: one produced by Watson (top) and the other by Mallinckrodt (bottom). Manufacturers are depicted in yellow, distributors are in blue, and final distributors are in light blue. The white arrows illustrate two possible distribution paths. The size of each circle is proportional to the annual volume shipped. For both manufacturers, one located in the eastern part and the other in the western region, we observe a significant concentration of distributors and final distributors, especially on the east side.

**Fig. 4:**
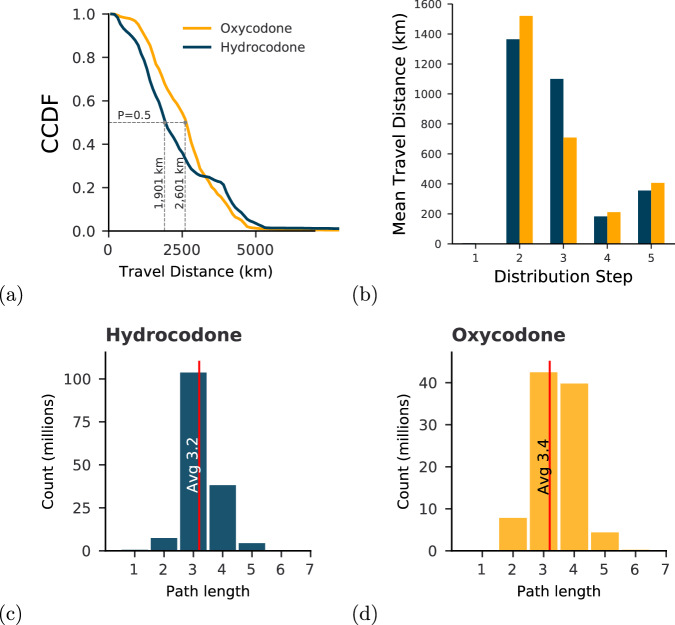
Probability distributions of travel distances and path lengths. (**a**) Cumulative Complementary Distribution Function (CCDF) of the distance traveled for oxycodone and hydrocodone from manufacturing to destination. The CCDF shows the probability that a package travels more than a given distance. (**b**) Mean travel distance for oxycodone and hydrocodone at each step of the distribution. (**c**) and (**d**) show the histograms of the path length for oxycodone and hydrocodone products and the average path length.

To further highlight the scale of this system, we compute the geographical distances traveled by packages along their distribution paths. Specifically, in Fig. 4(a), we show the total geographical distances traveled by hydrocodone and oxycodone products; and in Fig. 4(b), we show the average distance traveled by these products at each step of the distribution.

The cumulative complementary distribution (CCDF) shows the probability that a package travels more than a given distance. For example, 50% of hydrocodone packages travel at least 1,901 km, and oxycodone at least 2,601 km. This means that, on average, an oxycodone package travels 700km more than a hydrocodone package. Given that the east-west span of the contiguous US is roughly 4,500 km, it follows that an average hydrocodone/oxycodone package traverses nearly half of the country. Moreover, on average, the initial step of the distribution involves relatively short geographical distances. This suggests a close geographical proximity between manufacturers and first distributors. In fact, we found that most distributors connected to manufacturers have the same zip code as that manufacturer. The longest distance occurs during the second step, connecting the first distributor to the second one. Then, the geographical distances tend to decrease in the subsequent steps.

Interestingly, although the geographical distances are substantial, the path length (i.e., the number of distribution steps) is short. Most reconstructed paths have a length of three, meaning that products, starting from manufacturers, traverse two distributors before arriving at their final destination. This can also be seen in Fig. 4(c),(d) for oxycodone and hydrocodone-based products. Additionally, paths longer than five are rare, representing only 0.3% of all paths.

## Technical Validation

The dataset we provide is based on the assumption of a FIFO stock management strategy. To evaluate the impact of this assumption, we conduct a comparative analysis with data generated using the LIFO strategy.

Specifically, we evaluate the similarity of the paths reconstructed by both methods using the Jaccard similarity. This index measures the similarity between two sets (*A* and *B*) by comparing the ratio of their intersection to their union. Formally,

1 $${J}_{{\rm{Unique}}}(A,B)=\frac{| A\cap B| }{| A\cup B| }$$

This definition applies to sets with unique elements. For the case of distribution paths, this index can be used to assess how many unique paths both sets have in common. However, since the same paths may be used multiple times, we also calculate the weighted Jaccard similarity, which accounts for each path’s frequency. Formally,

2 $${J}_{{\rm{Weighted}}}(A,B)=\frac{{\sum }_{i}\min ({a}_{i},{b}_{i})}{{\sum }_{i}\max ({a}_{i},{b}_{i})}$$

where *a*_*i*_ and *b*_*i*_ are the multiplicity of element *i* in sets *A* and *B*, respectively.

**Fig. 5:**
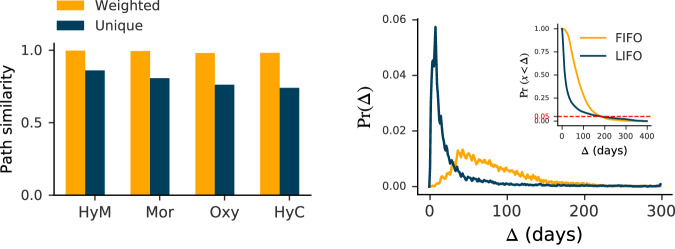
Comparison between LIFO and FIFO methods. (left) Shows the similarity for both unique and weighted Jaccard similarity, represented by green and yellow bars, respectively.(right) Features the Probability Density Function (PDF) corresponding to (Δ) days in transit under the two methods, and as an inset, the Complementary Cumulative Distribution Function (CCDF) is displayed, representing the probability for packages to be in transit for more than *x* days.

We find that the paths reconstructed by either method are highly similar. As depicted in Fig. 5 (left), the data reconstructed for four different active ingredients, using either LIFO or FIFO, show considerable overlap. When evaluating the unique paths using Eq. (1), the overlap is consistently above 74%. When considering the multiplicity of the paths, using Eq. (2), the similarity is always above 98%.

While the sequences of distributors traversed by packages remain similar under the LIFO assumption, the time in transit is different, as demonstrated in Fig. 5 (right). The LIFO approach yields paths with significantly shorter transit times for most delivered packages. However, approximately 5% of packages remain longer in stock compared to the FIFO strategy. This is illustrated as the switching point between the two cumulative distribution functions (CDFs) in the inset of Fig. 5 (right). Indeed, the FIFO strategy is recommended by the World Health Organization (WHO)^15^ and is most likely adopted for perishable goods with a finite shelf life, such as drugs.

As a second step in validation, we assess the similarity of the distribution paths from year to year. Given the dynamic nature of the market, it is reasonable to expect some variation; however, since the distribution of necessary drugs should be guaranteed, we do not expect sudden and large shifts.

**Fig. 6:**
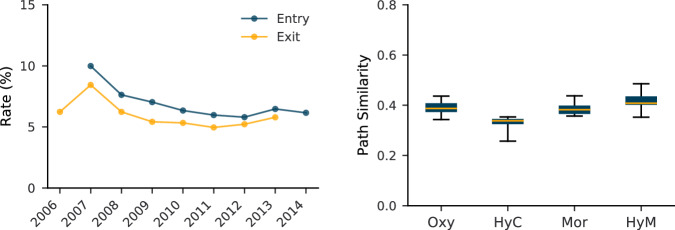
Entry and exit rate of distributors, and path stability. (left) Plot showing the entry and exit rates of distributors. This captures the dynamics of, for example, new manufacturers starting production, existing ones ceasing distribution, or pharmacy closures. (right) Box plot illustrating the year-to-year path similarity using the unique Jaccard similarity. In the box plot, the central rectangle spans the first quartile to the third quartile, the segment inside the rectangle shows the median, and the bars above and below show the minimum and maximum.

Figure 6 (left) shows the rate of entrance and exit of new distributors, i.e., a new manufacturer starts production, an existing warehouse ceases distribution, or a pharmacy closes down. We find that over the period covered by the dataset, entry and exit rates are between 5% and 10%. Note that the entry rate is always higher than the exit rate, which tells us that the number of distributors in the system increases.

Figure 6 (right) shows a moderate level of similarity across years, around 0.4. This can be attributed to the active market dynamics. Simultaneously, the error bars in the box plot (determined by the range between the maximum and minimum values) remain close to the average (indicated by the yellow line). This implies that while slight changes occur over the years, their magnitude remains consistent. This observation aligns with expectations for a pharmaceutical distribution system.

## Data Availability

The code used to generate the dataset is available at Figshare^16^.
